# Research Software vs. Research Data I: Towards a Research Data definition in the Open Science context

**DOI:** 10.12688/f1000research.78195.2

**Published:** 2022-11-01

**Authors:** Teresa Gomez-Diaz, Tomas Recio

**Affiliations:** 1 Laboraroire d'Informatique Gaspard-Monge, CNRS, Paris-Est, France; 2 Universidad Antonio de Nebrija, Madrid, Spain

**Keywords:** Research Data, Research Software, Open Science.

## Abstract

**Background: **Research Software is a concept that has been only recently clarified. In this paper we address the need for a similar enlightenment concerning the Research Data concept.

**Methods:** Our contribution begins by reviewing the Research Software definition, which includes the analysis of software as a legal concept, followed by the study of its production in the research environment and within the Open Science framework. Then we explore the challenges of a data definition and some of the Research Data definitions proposed in the literature.

**Results:** We propose a Research Data concept featuring three characteristics: the data should be produced (collected, processed, analyzed, shared & disseminated) to answer a scientific question, by a scientific team, and has yield a result published or disseminated in some article or scientific contribution of any kind.

**Conclusions:** The analysis of this definition and the context in which it is proposed provides some answers to the Borgman’s conundrum challenges, that is, which Research Data might be shared, by whom, with whom, under what conditions, why, and to what effects. They are completed with answers to the questions: how? and where?

## 1. Introduction

Each particle of the Universe, known or unknown by what is widely accepted as Science, is information. Different datasets can be associated to each particle to convey information, as, for example: where has this particle been discovered? By whom? At what time? Is this particle a constituent element of a rock, or a plant, or … ? Indeed, as living entities of the Earth planet, *… we are all part of this Universe and every atom in our bodies came from a star that exploded …* , therefore *… we are all stardust …* . ^1^

So long ago that we have never been able to give a precise date, information started to be fixed in cave paintings, figurines, and bone cravings, which have been found in caves like Altamira ^2^ or Lascaux ^3^. That is, some human beings *intentionally fixed information on a support.* Much more recently, languages have been developed to deal with information, fixing and exchanging it in clay bricks, papyrus, monument walls, and paper books. Even more recently, information has been fixed in films, photographs, and has finally adopted digital formats.

Scientists study all kinds of subjects and objects: persons, animals, trees and plants and other living beings, philosophies and philosophers, artists and artworks, mathematical theories, music, languages, societies, cities, Earth and many other planets and exoplanets, clouds, weather and climate, stars and galaxies, as well as other animate or inanimate objects, molecules, particles, nanoparticles and viruses, nowadays including digital objects such as computer programs. Some of these items, like images, texts, and music etc. may have associated intellectual property rights; but others, like statistics or geographical data, may not. Yet, they may be affected by other legal contexts, such as, for example, the one given by the EU INSPIRE Directive 1 for spatial data, concerning *any data with a direct or indirect reference to a specific location or geographical area.*

Now, in our digital era, most of the above subjects under consideration are handled by humans using computers, through numerical data. Scientists present new theories and results built and produced with numerical simulations and through the analysis of numerical datasets. They are usually stored in databases, manipulated or produced in digital environments using existing software, either Free/Open Source Software (FLOSS) ^4^ or commercial, or by means of software developed by research teams to address specific problems 2, 3.

In this specific scientific context, the aims and developments of Open Science practices are particularly relevant. Indeed, as remarked by 4: *"We must all accept that science is data and that data are science … ".* Therefore, in this article we take into consideration the following definition of Open Science, in which the open access to Research Data (RD) and to Research Software (RS) is part of the core pillars 5:

*Open Science is the political and legal framework where research outputs are shared and disseminated in order to be rendered visible, accessible and reusable.*

A more transversal and global vision can be found in the UNESCO Recommendation on Open Science ^5^, 6. See also 7 for another relevant example of ongoing work on the Open Science concept. But in this paper, following the analysis and the conclusions of 5, we focus here on this restricted framework as more suitable for our purposes.

Among the most important kinds of research outputs of any scientific work, we focus on the trio formed by *articles*, *software* and *data.* Actually, among all the possible duos, the couple RS and RD present more similarities, although a light list of differences between software and data have been mentioned in 8 and 9. On the other hand, regarding other duos, we think that differences are much stronger. For instance, unlike the dissemination of published articles, usually at the hands of scientific editors, the dissemination of software and data that have been produced in the research process is mostly at the hands of their producers, the research team. The analogies between RS and RD have been already summarily highlighted in 10, such as those concerning the release protocols of RD and RS, which raises the same questions, at the same time, in the production context. As a direct consequence, it seems suitable to propose a similar dissemination procedure for both kinds of research outputs 11.

Indeed, let us remark that, as mentioned in 11, 12, both RS and RD dissemination might involve the use of licenses to set their sharing conditions, such a core issue. Information about RS licenses and licensing can be found at the Free Software Foundation (FSF) ^6^, the Open Source Initiative (OSI) ^7^, and the Software Package Data Exchange (SPDX) ^8^. The SPDX licenses list also includes licenses that can be used for databases, like the Creative Commons licenses ^9^ or the Open Data Commons Licenses ^10^, see for example 13.

Other similarities regarding RS and RD are related to management plans: for example, Data Management Plans are nowadays required by research funders (see for example 14, 15) and, in the same mood, Software Management Plans have been recently proposed, see 16 and the references therein.

Finally, concerning evaluation, as observed in 3, similar evaluation protocols can be proposed for both RS and RD.

Leaving aside the common issues in RS and RD for licensing and management plans, that have been already studied in the above mentioned references, the RS and RD dissemination and evaluation analogies are more closely analyzed in the article 12 that follows the present work, including FAIR related issues 17 and 5Stars Open Data ^11^. On the other hand, in the current article we focus on the conceptual analogies of RS and RD, and their consequences (see Section 5).

As we will argue in the next sections, a definition for RD can be proposed following the main features of the RS definition given in our recent work 3, 18. However, we consider that formulating such proposal still remains a challenging issue that we dare to address here. In fact, although one of the most widely accepted RD definitions is the one proposed by the OECD (2007) 19, other works have shown the difficulties to fix such a definition 20, 21. Indeed, establishing this concept has important and not well settled consequences, for example, concerning the context of RD sharing, as highlighted by C. Borgman in 22:

*Data sharing is thus a conundrum. […]*

*The challenges are to understand which data might be shared, by whom, with whom, under what conditions, why, and to what effects. Answers will inform data policy and practice.*

It is the intention of our present work to bring some answers to these questions.

The plan of this article is as follows. The next section introduces the concept of RS after a summary presentation of the key points involved in the notion of software as a legal object. Section 3 is devoted to discuss the different issues involved in the challenge towards a precise definition of data (in the more comprehensive sense of this concept). Section 4 describes partially the landscape of existing work addressing the RD definition, enumerating, again, some difficulties to settle such a concept.

There we propose our RD definition, based in three characteristics: the data should be produced (collected, processed, analyzed, shared & disseminated) to answer a scientific question, by a scientific team, and has yield a result published or disseminated in some article or scientific contribution of any kind. Comparisons with other RD definitions are examined.

The last and final section concludes with the proposition of some specific answers to *Borgman’s conundrum challenges* 22. Let us remark that these conundrum challenges involve as well RD dissemination issues that are studied in detail in the article that follows this work 12, which also includes the analysis of RD evaluation and FAIR issues.

The reader of the current work should be aware that its authors are not legal experts. Thus, in order to address our goals in this article, we have analyzed (French, Spanish, European and USA) legal documents and articles written by law experts 1, 13, 20, 21, 23– 34, but from the *scientist’s point of view.* Yet, a deeper understanding of legal issues may require the intervention of legal specialists.

Following the standard scientific protocol, the authors of this work (mathematicians) have, first, detected a problem – the need to provide a more suitable RD definition. Then, they have observed the involved landscape and studied the related literature; have focused on and structured different components of the problem; finally, they have proposed what they believe could be a solution for the challenge under consideration. As in any other research work, we, authors of the present work, believe that our proposal should be examined by the scientific community in order to evaluate its correctness, and to help improving it, if needed, advancing towards a better solution.

## 2. Research Software

In this section we bring together some of the existing definitions of software as a legal object (see references below). We also recall our definition of RS coming from 3, 18.

### 2.1 Software is a legal object

In what follows we refer to the documents 26– 29 dealing with a definition of software as a legal object. Note that the terms *computer program*, *software*, *logiciel* (in French), *programa de ordenador* (in Spanish) are synonyms in this work. The terms *source code* (or *código fuente* in Spanish), *compiled code* (or *code compilé*, *código compilado*) correspond to subsets of a computer program.

The first definition that we would like to consider comes from the Directive 2009/24/EC of the European Parliament 26, that states:

*For the purpose of this Directive, the term “computer program” shall include programs in any form, including those which are incorporated into hardware. This term also includes preparatory design work leading to the development of a computer program provided that the nature of the preparatory work is such that a computer program can result from it at a later stage.*

Moreover, in the Spanish *Boletín Oficial del Estado* n. 97 (1996) 27 we can find ^12^:

*A los efectos de la presente Ley se entenderá por programa de ordenador toda secuencia de instrucciones o indicaciones destinadas a ser utilizadas, directa o indirectamente, en un sistema informático para realizar una función o una tarea o para obtener un resultado determinado, cualquiera que fuere su forma de expresión y fijación. […] comprenderá también su documentación preparatoria.*

*[For the purpose of this Law, a computer program shall be understood as any sequence of instructions or indications intended to be used, directly or indirectly, in a computer system to perform a function or a task or to obtain a certain result, whatever expression and fixation form it can take. […] it can also include its preparatory documentation.]*

Likewise, in the French *Journal officiel de la République française* (1982) 29 we can read:

***Logiciel** : Ensemble des programmes, procédés et règles, et éventuellement de la documentation, relatifs au fonctionnement d’un ensemble de traitement de données (en anglais : software).*

*[ **Software**: All programs, procedures and rules, and possibly documentation, related to the performance of some data processing (in English: software).].*

And in the French *Code de la propriété intellectuelle* (current regulation) 28, Article L112-2, we can find:

*Les logiciels, y compris le matériel de conception préparatoire, sont considérés notamment comme œuvres de l’esprit au sens du présent code.*

*[Software, including the preparatory material, is considered as works protected by the present code.]*

We observe that, in the above mentioned documents, the concept of software or computer program, *logiciel* or *programa de ordenador* refers to the set of instructions, of any kind, that are to be used in a computer system (including hardware). It is a work protected by the author rights. It can include the source code, the compiled code, and, eventually, the associated documentation and the preparatory material. It can be related to some data processing or to other tasks to be implemented in a computer system.

In order to complete this legal vision of the software concept we refer to item (11) of 26:

*For the avoidance of doubt, it has to be made clear that only the expression of a computer program is protected and that ideas and principles which underlie any element of a program, including those which underlie its interfaces, are not protected by copyright under this Directive. In accordance with this principle of copyright, to the extent that logic, algorithms and programming languages comprise ideas and principles, those ideas and principles are not protected under this Directive. In accordance with the legislation and case-law of the Member States and the international copyright conventions, the expression of those ideas and principles is to be protected by copyright.*

Indeed, there is a difference between the concepts of *algorithm* and *software* from the legal point of view, as there is a difference between the mere idea for the plot of a novel and the final written work. Several persons could have the same idea for the plot, but its realization in a final document will deliver different novels by different writers, as the novel will reflect the personality of its author. Similarly, an algorithm remains on the side of ideas, and as such, it is not protected by copyright laws. On the other side, poetry, novels and software are protected under copyright laws. Moreover, a computer program can implement several algorithms, and the same algorithm can be implemented in several programs.

Finally, note the nature of software as a digital object underlying all the above considerations.

### 2.2 Software as a research output: definition of Research Software

Beyond the vision of software as a legal object, we bring here the concept of Research Software (RS) as a scientific production, as defined in 3, 18:

***Research Software** is a well identified set of code that has been written by a (again, well identified) research team. It is software that has been built and used to produce a result published or disseminated in some article or scientific contribution. Each research software encloses a set (of files) that contains the source code and the compiled code. It can also include other elements as the documentation, specifications, use cases, a test suite, examples of input data and corresponding output data, and even preparatory material.*

Thus, Section 2.1 of 3 introduces several definitions regarding the notions of scientific and research software as found in the literature, as a way to support the above definition, while 18 provides complementary analysis on this concept. Note that this definition does not take into consideration if the RS status is “ongoing” or “finalized”, and does not regard if the RS has been disseminated, its quality or scope, its size, or if it is documented, maintained, used only by the development team for the production of an article, or it is currently used in several labs … 2.

Different recent works on the RS concept can be found, for example, on 35 and the references therein, where the *RDA FAIR for Research Software (FAIR4RS)* working group ^13^ proposes a definition of RS full of subtleties and details, albeit, perhaps, of complex interpretation in practice.

We observe, following our proposed definition, that RS can be characterized through three main features:

• the goal of the RS development is to do research. As stated by D. Kelly: *it is developed to answer a scientific question* 36,
• it has been written by a research team,
• the RS is involved in the obtention of the results presented in scientific articles (as the most important means for scientific exchange are still articles published in scientific journals) or by any other kind of recognized scientific means.

Note that documentation, licenses, examples, data, tests, Software Management Plans and other related information and materials can also be part of the set of files that constitutes a specific RS. Remark that the *data* we refer to in this list will qualify as RD (as defined in Section 4) if they have been produced by a research team, that can be the same team that has produced the RS, but not necessarily (notice that the role of the research team involved in the development of a RS has been thoroughly studied in Section 2.2 of 3). Indeed, Section 2.1 above shows that the preparatory design work and documentation are part of the software, and these are documents that can be included in the released version of a RS, following the choice of the RS producer team. There can be other elements as for example tests, input and output files to illustrate how to use the RS, licenses, etc. To include these elements in the released RS correspond to best practices that facilitate RS reuse. In our view, the release of a RD (see Section 4 and 12) can follow similar practices, that is, to include a documentation, some use examples, a license, a data management plan … this is to be decided by the producer team.

The initial origin of this RS definition is to be found in 2, that contains a detailed and complete study comparing articles and software produced in a typical (French) research lab. As remarked in received comments and Referee reports to this article, this RS definition (as well as the RD definition proposed in Section 4) is placed in what can be considered as a narrow context, emphasizing the role of the scientific production context. The relevance of such context is widely accepted by the scientific community in the case of articles: not every article published in a newspaper qualifies as a research article, that requires to be released in a scientific journal and subject to a referee procedure. Similarly, the importance of the production context has been already highlighted in the case of data, regarding those that qualify as cultural data 23.

Besides, our definition does not include as RS neither commercial software nor existing Free/Open Source Software (FLOSS) or other software developed outside Academia, a restriction which does not exclude that RS (or research articles, data...) can be produced in other contexts like private laboratories, for example. Rather, this means that we are not considering here differences between private or public funding of research. As a matter of fact, a research team can use RS produced by other teams for their scientific work, as well as FLOSS or other software developed outside the scientific community, but the present work is centered in the making-of aspects which are pertinent for the proposed definition. Obviously, a RS that has been initially developed in a research lab can evolve to become commercial software or just evolve outside its initial academic context. The above definition concerns its early, academic life.

Moreover, a RS development team may not just ***use*** software produced by other teams, but also ***include*** external software as a component *inside* the ongoing computer program, a procedure that could be facilitated by the FLOSS licenses. We consider that this external component qualifies as RS if it complies with the three characteristics given in the above definition. Moreover, the producers of the final RS should clearly identify the included external components, and their licenses. They should also highlight the used or included RS components, by means of a correct citation form 3, 8, 11, 37– 39.

Furthermore, a RS may ***involve*** other software components that can remain *external*, and that are not included in the RS development and release. It is then left to the users the task to recover and install them, and to assemble these external components in order to get a running environment. Another situation, as the one we have analyzed in 18, deals with the RS developed *within* a given software environment which is not perhaps fully disseminated with the RS. For example, the GeoGebra code developed by T. Recio and collaborators ^14^ does not disseminate the whole GeoGebra software ^15^, but only some parts that are relevant for their goals and that include their code.

See 2, 3, 18 for more discussions and references that have motivated the RS definition we have sketched in this section.

## 3. The challenges of a data definition

As stated in 40:

*“Data” is a difficult concept to define, as data may take many forms, both physical and digital.*

For example, unlike software, data is, as a legal object, much more difficult to grasp. In fact, according to 33, data is not a legal concept, as it does not fall into a specific legal regime. For example, data can be either mere information or *une œuvre*, a work with associated intellectual property, when it involves creative choices in its production that reflect the author’s personality 32. The Knowledge Exchange report 21 provides guidelines that can be used to assess the legal status of research data, and mentions:

*It is important to know the legal status of the data to be shared. […] not all data are protected by law, and not every use of protected research data requires the author’s consent. […] Whether data are in fact protected must be determined on a case-by-case basis.*

In relation with this legal context of data sharing and reuse, a very complete framework is introduced in 23:

*Les problématiques liées à la réutilisation nécessitent une maîtrise parfaite du droit de la propriété intellectuelle, du droit à l’image, du droit des données personnelles, du respect à la vie privée et du secret de la statistique, du droit des affaires, du droit de la concurrence, du droit de la culture, du droit européen et des règles de l’économie publique.*

*[The issues related to reuse require a perfect mastership of intellectual property rights, image rights, personal data rights, respect for private life and statistical confidentiality, business law, competition law, cultural law, European law and the rules of the public economy.]*

Another list of legal issues related to data is provided by 33, similar but not equal to the one in the previous quote. Yet, it is also necessary to consider other legal contexts concerning, for example, *les données couvertes par le secret médical ou le secret industriel et commercial [Data covered by medical secret or by the industrial and commercial secret]* ^16^. Let us remark that the section *Applicable Laws and Regulations* of 15 provides a broad overview of regulatory aspects that need to be taken into consideration when developing disciplinary RD management protocols in the European context. But, as declared in the introduction, it is not our intention to go deeper into these legal aspects, that should be also regarded from the perspective of many different laws.

The underlying problem is that data can refer to many different subjects or objects. We need to simplify the context to help us setting a manageable concept of research data adapted to the scientific framework. For this purpose we present here two relevant data definitions found in the data scientific literature.

The OECD data definition in its Glossary of Statistical Terms ^17^ states that:

**DATA**

*Definition: Characteristics or information, usually numerical, that are collected through observation.*

*Context: Data is the physical representation of information in a manner suitable for communication, interpretation, or processing by human beings or by automatic means (Economic Commission for Europe of the United Nations (UNECE)), “Terminology on Statistical Metadata”, Conference of European Statisticians Statistical Standards and Studies, No. 53, Geneva, 2000.*

Also, as a relevant precedent, let us quote here the data definition of the *Committee for a Study on Promoting Access to Scientific and Technical Data for the Public Interest*, as mentioned in 41:

*A data set is a collection of related data and information – generally numeric, word oriented, sound, and/or image – organized to permit search and retrieval or processing and reorganizing. Many data sets are resources from which specific data points, facts, or textual information is extracted for use in building a derivative data set or data product. A derivative data set, also called a value-added or transformative data set, is built from one or more preexisting data set(s) and frequently includes extractions from multiple data sets as well as original data (Committee for a Study on Promoting Access to Scientific and Technical Data for the Public Interest, 1999, p. 15).*

We can notice that both definitions combine the concepts of data and information, yielding, again, to a challenging situation. Thus, to better grasp the connection between both terms we have consulted several sources of different nature, see Box 1. Note that we can find in Box 1 that *information* among the data synonyms in the Larousse dictionary, but *data* is not among the information synonyms. On the other hand, Wikipedia mentions that both terms can be used interchangeably, but that they have different meanings.

Box 1. **A promenade around the data and information concepts.**

**I.1 *Diccionario de la lengua española* of the *Real Academia Española***

• Definition of *dato* ( https://dle.rae.es/dato)
– *Del latín datum ‘lo que se da’.*
– *1. m. Información sobre algo concreto que permite su conocimiento exacto o sirve para deducir las consecuencias derivadas de un hecho. A este problema le faltan datos numéricos.*
– *2. m. Documento, testimonio, fundamento.*
– *3. m. Inform. Información dispuesta de manera adecuada para su tratamiento por una computadora.*
• Definition of *información* ( https://dle.rae.es/informaci%C3%B3n)
– *Del latín informatio, -* $\bar{o}$ *nis ‘concepto’, ‘explicación de una palabra’.*
– *1. f. Acción y efecto de informar.*
– *2. f. Oficina donde se informa sobre algo.*
– *3. f. Averiguación jurídica y legal de un hecho o delito.*
– *4. f. Pruebas que se hacen de la calidad y circunstancias necesarias en una persona para un empleo u honor. U. m. en pl.*
– *5. f. Comunicación o adquisición de conocimientos que permiten ampliar o precisar los que se poseen sobre una materia determinada.*
– *6. f. Conocimientos comunicados o adquiridos mediante una información.*
– *7. f. Biol. Propiedad intrínseca de ciertos biopolímeros, como los ácidos nucleicos, originada por la secuencia de las unidades componentes.*
– *8. f. desus. Educación, instrucción.*

**I.2 *Diccionnaire Larousse de la langue française***

• Definition of *donnée* ( https://www.larousse.fr/dictionnaires/francais/donn%c3%a9e/26436)
– *Ce qui est connu ou admis comme tel, sur lequel on peut fonder un raisonnement, qui sert de point de départ pour une recherche (ex. Les données actuelles de la biologie).*
– *Idée fondamentale qui sert de point de départ, élément essentiel sur lequel est construit un ouvrage (ex. Les données d’une comédie).*
– *Renseignement qui sert de point d’appui (ex. Manquer de données pour faire une analyse approfondie).*
– *Représentation conventionnelle d’une information en vue de son traitement informatique.*
– *Dans un problème de mathématiques, hypothèse figurant dans l’énoncé.*
– *Résultats d’observations ou d’expériences faites délibérément ou à l’occasion d’autres tâches et soumis aux méthodes statistiques.*
• Definition of *information* ( https://www.larousse.fr/dictionnaires/francais/information/42993)
– *Action d’informer quelqu’un, un groupe, de le tenir au courant des événements : La presse est un moyen d’information.*
– *Indication, renseignement, précision que l’on donne ou que l’on obtient sur quelqu’un ou quelque chose: Manquer d’informations sur les causes d’un accident. (Abréviation familière : info.)*
– *Tout événement, tout fait, tout jugement porté à la connaissance d’un public plus ou moins large, sous forme d’images, de textes, de discours, de sons. (Abréviation familière : info.)*
– *Nouvelle communiquée par une agence de presse, un journal, la radio, la télévision. (Abréviation familière : info.)*
– ***Cybernétique.** Mesure de la diversité des choix dans un répertoire de messages possibles.*
– ***Droit.** Instruction préparatoire, diligentée par le juge d’instruction en vue de rechercher et de rassembler les preuves d’une infraction, de découvrir l’auteur, de constituer à charge et à décharge le dossier du procès pénal. (Elle est close par un non-lieu ou par un renvoi devant une juridiction répressive. En matière criminelle, l’instruction est à double degré [juge d’instruction, chambre d’accusation].)*
– ***Informatique.** Élément de connaissance susceptible d’être représenté à l’aide de conventions pour être conservé, traité ou communiqué.*

**I.3 *Wikipedia***

Extract from the *Data* page of Wikipedia ( https://en.wikipedia.org/wiki/Data):

*Data are characteristics or information, usually numeric, that are collected through observation. In a more technical sense, data are a set of values of qualitative or quantitative variables about one or more persons or objects, while a datum (singular of data) is a single value of a single variable.*

*[…]*

*Although the terms “data” and “information” are often used interchangeably, these terms have distinct meanings. […] data are sometimes said to be transformed into information when they are viewed in context or in post-analysis. However, […] data are simply units of information.*

Moreover, in 42 and in the web page of ISKO ^18^, when discussing in detail the concept of data, an etymological and linguistic vision is also the starting point, and among other sources also, it mentions Wikipedia. The conclusion in 42 (section 2.5):

*Therefore, our conclusion of this Section is that Kaase’s (2001, 3251) definition seems the most fruitful one suggested thus far:*

*Data are information on properties of units of analysis.*

See also 43– 45 where ours readers can find further reflections on the concepts of data, information, knowledge, understanding, evidence and wisdom.

Such reflections bring to us an eclectic panorama on the ingredients that could form a data definition and their relation with the concept of information, attesting the involved difficulties in such goal.

Focusing in the scientific context, we can illustrate this complexity in full terms referring to the French *Code de l’environnement* 30. In its Article L-124-2 ^19^ we can appreciate the subtleties of the definition of environmental data in the following description:

*Est considérée comme information relative à l’environnement au sens du présent chapitre toute information disponible, quel qu’en soit le support, qui a pour objet :*

***1.** L’état des éléments de l’environnement, notamment l’air, l’atmosphère, l’eau, le sol, les terres, les paysages, les sites naturels, les zones côtières ou marines et la diversité biologique, ainsi que les interactions entre ces éléments ;*

***2.** Les décisions, les activités et les facteurs, notamment les substances, l’énergie, le bruit, les rayonnements, les déchets, les émissions, les déversements et autres rejets, susceptibles d’avoir des incidences sur l’état des éléments visés au point 1 ;*

***3.** L’état de la santé humaine, la sécurité et les conditions de vie des personnes, les constructions et le patrimoine culturel, dans la mesure où ils sont ou peuvent être altérés par des éléments de l’environnement, des décisions, des activités ou des facteurs mentionnés ci-dessus ;*

***4.** Les analyses des coûts et avantages ainsi que les hypothèses économiques utilisées dans le cadre des décisions et activités visées au point 2 ;*

***5.** Les rapports établis par les autorités publiques ou pour leur compte sur l’application des dispositions législatives et réglementaires relatives à l’environnement.*

*[For the purposes of this chapter, information relating to the environment is considered to be any information available, whatever the medium, the purpose of which is:*

***1.** The state of the elements of the environment, namely the air, atmosphere, water, soil, land, landscapes, natural sites, coastal or marine areas and biological diversity, as well as the interactions between these elements;*

***2.** Decisions, activities and factors, namely substances, energy, noise, radiation, waste, emissions, spills and other discharges, likely to have an impact on the state of the elements concerned in point 1;*

***3.** The state of human health, safety and living conditions of people, buildings and cultural heritage, insofar as they are or may be altered by elements of the environment, decisions, activities or the factors mentioned above;*

***4.** The analyses of costs and advantages as well as the economic assumptions used in the context of the decisions and activities referred to in point 2;*

***5.**Reports drawn up by public authorities or on their behalf on the application of legislative and regulatory provisions related to the environment.*] *.*

To be compared with the much more easier to understand concept of geographical data as introduced by the Article L127-1 ^20^ of the same *Code de l’environnement* 30:

*Donnée géographique, toute donnée faisant directement ou indirectement référence à un lieu spécifique ou une zone géographique* ;

*[Geographic data, any data that refers directly or indirectly to a specific place or geographic area;]*

Another example to show the complexity of the representation and manipulation of data and information that we would like to mention here corresponds to the linguistic research work developed at the Laboratoire d’informatique Gaspard-Monge, where one of the authors of the present work resides, see for example the doctoral thesis 46, 47.

An additional factor that adds complexity to the concept of scientific data has to do with the potential use(s) and sharing of these data. As remarked by the OECD Glossary of Statistical Terms ^21^:

*The context provides detailed background information about the definition, its relevance, and in the case of data element definitions, the appropriate use(s) of the element described.*

The importance of the context is also noted in 22:

*… research data take many forms, are handled in many ways, using many approaches, and often are difficult to interpret once removed from their initial context.*

This opens the door to a series of complex issues. For example, to the need for complementary, technical information or documentation associated to a given dataset in order to facilitate its reuse. See 48 (p.16) (and also 40) that highlights the difficulties raised by the concept of *temperature* related data, as explained by a CENS biologist:

*There are hundreds of ways to measure temperature. “The temperature is 98” is low-value compared to, “the temperature of the surface, measured by the infrared thermopile, model number XYZ, is 98.” That means it is measuring a proxy for a temperature, rather than being in contact with a probe, and it is measuring from a distance. The accuracy is plus or minus.05 of a degree. I [also] want to know that it was taken outside versus inside a controlled environment, how long it had been in place, and the last time it was calibrated, which might tell me whether it has drifted.*

Another instance to further illustrate the complexity of technical information associated to a data set in the STRENDA Guidelines that have been developed to assist authors to provide data describing their investigations of enzyme activities. ^22^

Other examples from the collection of complex issues associated to data use(s) and sharing conditions are:

• 23 (p.11) The concept of *right of access*, involving the meaning of public information, requiring three characteristics: the existence of a document, of administrative nature, and in the possession of the Public Administration.
• 23 (p.13) The idea of *reuse*:
*… l’utilisation d’une information publique par toute personne qui le souhaite à d’autres fins que celles de la mission de service public pour les besoins de laquelle les documents ont été élaborés ou détenus.*
*[… the use of public information by anyone who wishes it for other purposes than those of the original needs for which the documents were prepared or held by the public service mission.].*

finds a strong formulation for scientific data in 49:

*The value of data lies in their use. Full and open access to scientific data should be adopted as the international norm for the exchange of scientific data derived from publicly funded research. The public-good interests in the full and open access to and use of scientific data need to be balanced against legitimate concerns for the protection of national security, individual privacy, and intellectual property.*

For more information on ‘re-use’ see, for example, 20, 25, 32, 48.

• 23 (p.10) The evolution from the *right of access to documents from the Public Administration* to the *right of reuse of public information.*
• 23 (section II) The meaning of *free/libre reuse* of public information, under three circumstances:
1
public information derived from a document produced or hold by the Administration,
2
there are no other intellectual property rights owners,
3
data do not affect personal or private issues of people.
• 22 (p. 1060) The concept of *data sharing* in a scientific context:

*For the purposes of this article, data sharing is the release of research data for use by others. Release may take many forms, from private exchange upon request to deposit in a public data collection. Posting datasets on a public website or providing them to a journal as supplementary materials also qualifies as sharing.*

• The importance of licenses to set the *sharing* and *re-use* conditions as highlighted in 5, 11, 13, 50.
• The concepts of *Open Data* ^23^ and *Open access to data*, see for example 25, 32, 51– 53. As we can find in 25 and 53:
*Open data are data in an open format that can be freely used, re-used and shared by anyone for any purpose.*
• 53 also provides a classification of scientific data in four types: observational, experimental, computational and reference data sets.
• The FAIR guiding principles 17 are studied in the article that follows this work 12.
• The recent and relevant introduction of the term *Big Data* ^24^, that refers to the exploitation of larger amounts of data. They can appear in medical research, meteorology, genomics, astronomy, demographic studies … and in real life, as we live all in a digital world where we generate large amounts of data every day by the use of phones and computers to do work, travelling, e-mail, business, shopping etc. 42. Big data is associated mainly to four “V” characteristics: Volume, Variety, Velocity, Veracity, and others can be found for example in the mentioned Wikipedia page and in the references mentioned there. See also 54.

Closing the conceptual loop developed in this section, let us remark, again, that legal aspects arise quite naturally in the above list of items. Among others, some aspects are related to the fact that the datasets are usually organized in databases, where data is arranged in a systematic or methodical way and is individually accessible by electronic or other means 13, 20, 21, 24, 28. The intellectual property rights can apply to the content of a database, the disposition of its elements and to the tools that make it working (for example software). The *sui generis* database rights primarily protects the producer of the database and may prohibit, for instance, the extraction and/or reuse of all or a substantial part of its content 24.

Finally, let us quote here this paragraph from the OpenAIRE project report 20 (p.19) that highlights the difficulties to set a research data definition in the context of legal studies:

*From a legal point of view, one of the very basic questions of this study is which kind of potentially protected data we are dealing with in the context of e-infrastructures for publications and research data such as OpenAIREplus. The term “research data” in this context does not seem to be very helpful, since there is no common definition of what research data basically is.*

*It seems rather that every author or research study in this context uses its own definition of the term. Therefore, the term “research data” will not be strictly defined, but will include any kind of data produced in the course of scientific research, such as databases of raw data, tables, graphics, pictures or whatever else.*

We can remark, that although the preceding quote does not provide a strict definition of research data, it highlights the relevance of the production context, as we have already mentioned in Section 2.2.

## 4. Data as a research output: towards a definition for Research Data

In the previous section we have exemplified the complexity of the concept of data through different approaches. In this section we focus on the research data concept, proposing here a RD definition, directly derived from the RS definition presented in Section 2.2. To this aim we start by gathering some previous definitions that are particularly relevant for our proposal.

The first one is the White House document 34, and in particular the *Intangible property* section where we can find the following definition.

*Research data is defined as the recorded factual material commonly accepted in the scientific community as necessary to validate research findings, but not any of the following: preliminary analyses, drafts of scientific papers, plans for future research, peer reviews, or communications with colleagues.*

Let us remark that, according to 34 this definition explicitly excludes:

(A) *Trade secrets, commercial information, materials necessary to be held confidential by a researcher until they are published, or similar information which is protected under law; and*

(B) *Personnel and medical information and similar information the disclosure of which would constitute a clearly unwarranted invasion of personal privacy, such as information that could be used to identify a particular person in a research study.*

The above RD definition has been extended in 55, emphasizing, among other aspects, the scientific purpose of the recorded factual material and the link with the scientific community.

A second basic inspiration for our proposal is the Directive for Open Data 25 that states:

*(Article 2 (27)) The volume of research data generated is growing exponentially and has potential for re-use beyond the scientific community. […] Research data includes statistics, results of experiments, measurements, observations resulting from fieldwork, survey results, interview recordings and images. It also includes meta-data, specifications and other digital objects. Research data is different from scientific articles reporting and commenting on findings resulting from their scientific research.*

*[…]*

*(Article 2 (9)) ‘research data’ means documents in a digital form, other than scientific publications, which are collected or produced in the course of scientific research activities and are used as evidence in the research process, or are commonly accepted in the research community as necessary to validate research findings and results;*

The third pillar that we consider essential to support our proposal is the OECD report 19 (p.13) where we can find one of the most largely accepted and adopted definitions of RD:

*Research data are defined as factual records (numerical scores, textual records, images and sounds) used as primary sources for scientific research, and that are commonly accepted in the scientific community as necessary to validate research findings. A research data set constitutes a systematic, partial representation of the subject being investigated.*

*This term does not cover the following: laboratory notebooks, pre-liminary analyses, and drafts of scientific papers, plans for future research, peer reviews, or personal communications with colleagues or physical objects (e.g. laboratory samples, strains of bacteria and test animals such as mice). Access to all of these products or outcomes of research is governed by different considerations than those dealt with here.*

Finally, let us bring here the research data definition coming from the “Concordat on Open Research Data” ^25^ signed by the research councils of the UK Research and Innovation (UKRI) organisation ^26^ :

***Research data** are the evidence that underpins the answer to the research question, and can be used to validate findings regardless of its form (e.g. print, digital, or physical). These might be quantitative information or qualitative statements collected by researchers in the course of their work by experimentation, observation, modelling, interview or other methods, or information derived from existing evidence. Data may be raw or primary (e.g. direct from measurement or collection) or derived from primary data for subsequent analysis or interpretation (e.g. cleaned up or as an extract from a larger data set), or derived from existing sources here the rights may be held by others.*

Let us observe that this last definition highlights the important role of data as a tool to find an answer to a scientific question, coinciding with the first characteristic of our RS definition, and also agreeing with 40 (p. 508): … *data from scientific sensors are a means and not an end for their own research.*

A remarkable “positive” aspect of these four definitions is that they separate the data from the subject under study, and establish what is, or is not, RD. This is relevant, as the legal context of the subjects under study sets up the legal (and *ethical*) context of the RD.

We must say that we do not agree completely with all the terms in these definitions. For example, regarding the exclusion of the laboratory notebooks as RD elements, as we think they can be used to generate input data for other studies (how a laboratory works, which is the information that appears in some notebooks depending on the scientific matter). We think that these information and data can be of interest for other researchers.

Some other “negative” aspects: the role of the data producers does not appear in the above definitions, although it is more or less implicit when they refer to the connection with the scientific community. Indeed, their role is very important as observed in 48 (p.6):

*Data creators usually have the most intimate knowledge about a given dataset, gained while designing, collecting, processing, analyzing and interpreting the data. Many individuals may participate in data creation, hence knowledge may be distributed among multiple parties over time.*

Certainly, as for each research output, the producer team is the guarantor of the data quality, in particular to ensure that the data are not outdated, erroneous, falsified, irrelevant, and unusable. Note that this is particularly relevant in the case of RD, as a consequence of the lack of a widely accepted RD publication procedures, compared to the existing ones for articles in scientific journals, where the responsibility of the quality of the publication is somehow shared by the authors, the journal editors, and the reviewers. This is also confirmed by 56 (p. 73):

*The concept of data quality is determined by multiple factors. The first is trust. This factor is complex in itself. […] Giarlo (2013) also mentions trust in first place, stating that it depends on subjective judgments on authenticity, acceptability or applicability of the data. Trust is also influenced by the given subject discipline, the reputation of those responsible for the creation of the data, and the biases of the persons who are evaluating the data.*

Even more, note that, as remarked in 23 *the quality of the producer legal entity* defines the cultural quality of the data in legal terms, yielding in this way the qualification of *cultural data.*

On the other hand, in some of the above definitions, the RD scientific purpose is focused in its role to *validate research findings*, although RD can be reused for many other finalities in the scientific context as, for instance, to generate new knowledge, i.e. as primary sources for *new* scientific findings. Let us observe that these are two of the four rationales for data sharing examined in 22.

Bearing all these arguments in mind, we propose the following RD definition.

***Research data** is a well identified set of data that has been produced (collected, processed, analyzed, shared & disseminated) by a (again, well identified) research team. The data has been collected, processed and analyzed to produce a result published or disseminated in some article or scientific contribution. Each research data encloses a set (of files) that contains the dataset maybe organized as a database, and it can also include other elements as the documentation, specifications, use cases, and any other useful material as provenance information, instrument information, etc. It can include the research software that has been developed to manipulate the dataset (from short scripts to research software of larger size) or give the references to the software that is necessary to manipulate the data (developed or not in an academic context).*

We can summarize the above definition in the following three main characteristics:

• the goal of the collection and analysis is to do research, that is, to answer a scientific question (which includes the validation of research findings),
• it has been produced by a research team,
• the RD is involved in the obtention of the results presented in scientific articles (as the most important means for scientific exchange are still articles published in scientific journals) or by any other kind of recognized scientific means.

We provide here some further considerations concerning this proposal. First, it is clear that we have followed closely the RS definition in Section 2.2, in order to formulate this RD counterpart, which involves the transaltion of some RS features of strict *digital nature* to RD. This does not mean that we do not consider non digital data as possible RD, but rather we assume that the information extracted from the physical samples has been already treated as digital information to be manipulated in a computer system, which simplifies the manipulation of physical data and its inclusion in the proposed RD definition.

Secondly, we emphasize that our RD definition also follows the consideration of a restricted research production context, as in the case of our RS definition. But this limited context to set the RD definition does not mean that e.g. public sector data can not be used in the research work. Rather it means that the external components that have not been directly collected/produced by the research team should be well identified, indicating their origin, where the data is available, which is the license that allows the reuse. It is also necessary to indicate if the data has been reused (processed) without modification, or if some adaptations have been necessary for the analysis. External data components can have any origin, not just public sector. As we have highlighted in Section 3, the production context of the data may have a lot of importance, as data can be difficult to interpret once removed from their initial context 22.

Third, note that, according to our definition, documentation, licenses, Data Management Plans and other documents can also be part of the set of files that constitutes the RD. Moreover, as explained in Section 2.2, a RS can also include data in the list of included materials that could also be qualified as RD. There are here a broad spectrum of possibilities, according to the size, the importance given by the research team and the chosen strategy in the dissemination stage. If the RD is considered of little size and less importance than the RS, it can be just included and disseminated as part of the software, and also the other way around, when the RS is considered less important than the RD, as for example when the software development effort is much less important than the time and effort invested in the data collection and analysis. It can also happen that both outputs are considered as of equal value, and can be disseminated separately. In this case it is important that both outputs are linked in order to allow other researchers to find easily the other output.

In a similar manner as for RS, RD can include other data components, and some can also qualify as research data. The RD producer team should explain how these components have been selected, mixed and analyzed, and highlight the reuse of other RD components by means of a correct citation form, see for example, 38, 41, 57.

Moreover, software and data can have several versions and releases, and they can be manipulated alike and with similar tools (forges, etc …) 37, 58, 59. One of the differences that we have detected between RS and RD is that while some research teams can decide to give access to early stages of the software development, what we observe in the consulted work is that RD is expected in its final form, ready for reuse, as mentioned in 22:

*If the rewards of the data deluge are to be reaped, then researchers who produce those data must share them, and do so in such a way that the data are interpretable and reusable by others.*

This difference is a consequence of the distinct nature of the building process of both objects. In the FLOSS community, we find the *release early, release often* principle associated to the development of the Linux kernel 60 and to Agile developments. ^27^ This principle may not have the same sense in the building of a dataset for which a research team collects, processes and analyzes data with a very particular research purpose, maybe difficult to share with a large or external community in the early stages of the RD production.

Yet, in this work, we do not address some production issues like best software development practices or data curation, as they are out of the scope of the present article, and could be the object of future work. It is not that we do not give enough appreciation to these important issues, as they are part of the 3rd step of the proposed CDUR evaluation protocol for RS and RD, see sections 2.3 and 3.3 of 12. For us, the research team decides when the research outputs have reached the right status for its dissemination. Neither we do enter in the different roles (see 22) that may appear in the RD team, taking care of actions involving: collection, cleaning, selection, documentation, analysis, curation, preservation, maintenance, or the role of Data Officer proposed in 15.

## 5. Conclusion

While some authors highlight differences between software and data 8, 9, the present article leans toward profiting from the similarities shared by RS and RD. For example, taking into consideration the difference between the definition of software and the definition of RS has driven us to the proposition of a RD definition that is independent from the definition of data. Likewise, along the above sections we have emphasized other characteristics of RD that are grounded in the RS features. As a side effect of this approach, the fact that we can easily adopt issues from the RS definition formulation to RD, confirms and validates our proposed RS definition.

In the introduction we have mentioned Borgman’s conundrum challenges related to RD 22:

*The challenges are to understand which data might be shared, by whom, with whom, under what conditions, why, and to what effects. Answers will inform data policy and practice.*

In our experience, Borgman's conundrum challenges correspond to questions that appear regularly at different stages of the RD production. We think that to provide the vision developed in Section 4 could be of help to deal with these questions, as a first step to tackle some problems in a well determined situation. Moreover, the view proposed in Section 4 is extended and completed with the dissemination and evaluation protocols of 12. Our experience of many years confirms the need of these protocols for RS, and we think that they will be appropriated, useful and relevant for RD as well.

As a test for the soundness of the proposed RD definition we have used the conundrum queries as a benchmark, checking if our definition allows us to provide answers to the different questions, as well as to two extra ones that we consider equally relevant, namely **how** and **where** to share RD:

**Which data might be shared?** Following the arguments supporting our RD definition, we think that it is a decision of the research team: similarly to the stage in which the team decides to present some research work in the form of a document for its dissemination as a preprint, or a journal article, a conference paper, a book … the team should decide which data might be shared, in which form and when (following maybe funder or institutional Open Science requirements).

**By whom?** The research team that has collected, processed, analyzed the RD, and decided to share and disseminate it. That is the RD producer team, as stated in the second characteristic of our RD definition. On the other hand, data ownership issues have been discussed for example in 20, 21, 32, 61– 63.

**How?** As observed in the precedent sections, the How? should follow some kind of dissemination procedure like the one proposed in 11, 12 in order to identify correctly the RD set of files, to set a title and the list of persons in the producer team (that can be completed with their different roles), to determine the important versions and associated dates, to give a documentation, to verify the legal 21, 33 (and *ethical*) context of the RD and give the license to settle the sharing conditions 13, etc. which can include the publication of a data paper and decisions about in which form and when the RD should be disseminated, maybe following grant funders or institutional Open Science requirements). In order to increase the return on public investments in scientific research, RD dissemination could respect principles and follow guidelines as described in 17, 19. Further analysis on RD dissemination issues can be found in 12.

**Where?** There are different places to disseminate a RD, including the web pages of the producer team, of the funded project, or in a existing data repository. Let us remark that the Registry of Research Data Repository ^28^ is *a global registry of RD repositories that covers repositories from different academic disciplines.* It is funded by the German Research Foundation (DFG) ^29^ and it can help to find the right repository. Note that the Science Europe report 64 provides criteria for the selection of trustworthy repositories to deposit RD.

**With whom?** Each act of scholar communication has its own target public, and initially, the RD dissemination strategy can target the same public as the one that could be interested in the corresponding research article. But it can happen that the RD is of interdisciplinary value, possibly wider than the initial discipline associated to the scientific publication, and to assess what is the public involved in this larger context can be difficult. Indeed, as observed by 22:

*An investigator may be part of multiple, overlapping communities of interest, each of which may have different notions of what are data and different data practices. The boundaries of communities of interest are neither clear nor stable.*

So, it can be complex to determine the community of interest for a particular RD, but this also happens for articles, see for example the studies on HIV/AIDS 65 making reference to automatic reasoning in elementary geometry in studies in its reference number 12, and it seems to us that this has never been an obstacle for sharing a publication. Thus 22:

*… the intended users may vary from researchers within a narrow specialty to the general public.*

**Under what conditions?** As described previously, and in parallel with the case of RS, the sharing conditions are to be found in the license that goes with the RD, such as a Creative Commons license ^30^ or other licenses to settle the attribution, re-use, mining … conditions 13. For example, in France, the law of 2016 for a Digital Republic Act sets in a *Décret* the list of licenses that can be used for RS or RD release 31, 32.

**Why and to what effects?** There maybe different reasons to release some RD, from the contribution to build more solid, and easy to validate science, to just comply with the recommendations or requirements of the funder of a project, of the institutions supporting the research team, or those of a scientific journal, including Open Science issues 5. The works 22, 49 give a thorough analysis on this subject. As documented there and already mentioned in Section 3:

*“The value of data lies in their use. Full and open access to scientific data should be adopted as the international norm for the exchange of scientific data derived from publicly funded research.”*

As remarked in 5 and in the work analyzed there, the evaluation step is an important enabler in order to improve the adoption of Open Science best practices and to increase RD sharing and open access. To disseminate high quality RD outputs is a task that requires time, work and hands willing to verify the quality of the data, to write the associated documentation, etc. Incentives are needed to motivate the teams to accomplish these tasks. RD dissemination also asks for the establishment of best citation practices and evolution in the protocols of research evaluation. In particular, following the parallelism present all along this work, the CDUR protocol 3 proposed for RS evaluation can be also proposed for RD as developed in the article that extends the present work 12.

## Data availability

### Underlying data

Data underlying the arguments presented in this article can be found in the references, footnotes and Box 1.

## Footnotes

^1^
“We Are Star Dust” - Symphony of Science, https://www.youtube.com/watch?v=8g4d-rnhuSg

^2^
Cave of Altamira and Paleolithic Cave Art of Northern Spain, https://whc.unesco.org/en/list/310/

^3^
Prehistoric Sites and Decorated Caves of the Vézère Valley, https://whc.unesco.org/en/list/85/

^4^

https://en.wikipedia.org/wiki/Free_and_open-source_software

^5^

https://en.unesco.org/science-sustainable-future/open-science/recommendation

^6^

https://www.fsf.org/licensing/

^7^

https://opensource.org/licenses

^8^

https://spdx.org/licenses/

^9^

https://creativecommons.org/licenses/?lang=en

^10^

https://opendatacommons.org/licenses/

^11^

https://5stardata.info/en/

^12^
Note that the authors of this article provide their own translations. Authors prefer to keep the original text for two reasons. First, because of the legal nature of the involved quotations. Second, for French or Spanish speaking readers to enjoy it, very much in line with the Helsinki Initiative on Multilingualism in Scholarly Communication (2019), see https://doi.org/10.6084/m9.figshare.7887059. These translations have been helped by Google Translate, https://translate.google.com/ and Linguee, https://www.linguee.fr/.

^13^

https://www.rd-alliance.org/groups/fair-research-software-fair4rs-wg

^14^

https://matek.hu/zoltan/issac-2021.php

^15^

https://swmath.org/software/4203

^16^
See, for example, https://www.senat.fr/dossier-legislatif/pjl16-504.html

^17^

https://stats.oecd.org/glossary/detail.asp?ID=532

^18^

https://www.isko.org/cyclo/data

^19^

https://www.legifrance.gouv.fr/codes/article_lc/LEGIARTI000006832922/

^20^

https://www.legifrance.gouv.fr/codes/section_lc/LEGITEXT000006074220/LEGISCTA000022936254/

^21^
The entries of the glossary https://stats.oecd.org/glossary/ have several parts including *Definition* and *Context* as shown in the Data definition included in Section 3. This quotation appears when placing the pointer over the *Context* part of the Data entry.

^22^

https://www.beilstein-institut.de/en/projects/strenda/guidelines/

^23^

https://en.wikipedia.org/wiki/Open_data

^24^

https://en.wikipedia.org/wiki/Big_data

^25^

https://www.ukri.org/wp-content/uploads/2020/10/UKRI-020920-ConcordatonOpenResearchData.pdf

^26^

https://www.ukri.org/

^27^

https://en.wikipedia.org/wiki/Agile_software_development

^28^

https://www.re3data.org/

^29^

http://www.dfg.de/

^30^

https://creativecommons.org/
