# Supplementary material for: Research Software vs. Research Data I: Towards a Research Data definition in the Open Science context
Source: F1000Res. 2022 Nov 1;11:118. Originally published 2022 Jan 28. [Version 2] doi: 10.12688/f1000research.78195.2 (PMC9650106; doi:10.12688/f1000research.78195.2)
Supplement: Peer review history

# Peer review: Reviewer response for version 2

**DOI:** 10.5256/f1000research.138878.r154717

**Published:** 2022-12-09

**Authors:** Joachim Schopfel

**Affiliations:** 1 GERiiCO Labor, University of Lille, Lille, France

The second version is fine with me. The authors replied to all comments; they fixed some issues, and they provided complementary arguments for other issues. I do not share all their viewpoints but that is science and not a problem. The paper is interesting and relevant.

Is the work clearly and accurately presented and does it cite the current literature?

Partly

If applicable, is the statistical analysis and its interpretation appropriate?

Not applicable

Are all the source data underlying the results available to ensure full reproducibility?

No source data required

Is the study design appropriate and is the work technically sound?

Partly

Are the conclusions drawn adequately supported by the results?

Partly

Are sufficient details of methods and analysis provided to allow replication by others?

Partly

Reviewer Expertise:

Information science

I confirm that I have read this submission and believe that I have an appropriate level of expertise to confirm that it is of an acceptable scientific standard.

# Peer review: Reviewer response for version 2

**DOI:** 10.5256/f1000research.138878.r154719

**Published:** 2022-12-02

**Authors:** Remedios Melero

**Affiliations:** 1 Instituto de Agroquímica y Tecnología de Alimentos, CSIC, Valencia, Spain

I do not have any further comments.

Is the work clearly and accurately presented and does it cite the current literature?

Partly

If applicable, is the statistical analysis and its interpretation appropriate?

Not applicable

Are all the source data underlying the results available to ensure full reproducibility?

No source data required

Is the study design appropriate and is the work technically sound?

Partly

Are the conclusions drawn adequately supported by the results?

Partly

Are sufficient details of methods and analysis provided to allow replication by others?

Partly

Reviewer Expertise:

Open science, open research data, scholarly publications, open access policies

I confirm that I have read this submission and believe that I have an appropriate level of expertise to confirm that it is of an acceptable scientific standard.

# Peer review: Reviewer response for version 2

**DOI:** 10.5256/f1000research.138878.r154718

**Published:** 2022-11-10

**Authors:** Tibor Koltay

**Affiliations:** 1 Institute of Learning Technologies, Eszterházy Károly University, Eger, Hungary

I am satisfied with the author’s reply, and found the other two reviews’ comments intriguing and useful for the authors. I have no further comments.

Is the work clearly and accurately presented and does it cite the current literature?

Partly

If applicable, is the statistical analysis and its interpretation appropriate?

Not applicable

Are all the source data underlying the results available to ensure full reproducibility?

No source data required

Is the study design appropriate and is the work technically sound?

Partly

Are the conclusions drawn adequately supported by the results?

Partly

Reviewer Expertise:

Information science

I confirm that I have read this submission and believe that I have an appropriate level of expertise to confirm that it is of an acceptable scientific standard.

# Peer review: Reviewer response for version 1

**DOI:** 10.5256/f1000research.82187.r121514

**Published:** 2022-04-19

**Authors:** Joachim Schopfel

**Affiliations:** 1 GERiiCO Labor, University of Lille, Lille, France

The research data management is a central dimension of the development of scientific research and related infrastructures. Also, any original attempt to define research data is welcome and helpful for the understanding of this field. This conceptual paper will be a valuable contribution to the discussion on the research but. Yet, it should be improved, and a couple of more or less minor issues should be fixed.

1. First, all cited text should be systematically translated into English.
2. The main concepts (such as data, information, knowledge...) should be defined from the beginning on and not only later (section 3). The definitions should not be based on Wikipedia, Larrousse etc but on academic works in the field of information science (eg ISKO). 
3. Open science is a fuzzy concept, an umbrella term or even a "boundary object" (as Samuel Moore put it). But it should be made clear that open science is more than "sharing and dissemination of research outputs" (as in the [5] citation). 
4. The former comment is important because the approach of the paper is in some kind limited or reduced to the aspect of "research output". Generally, in the research process, research software and research data are not only output but also tools (software) and input (data). This needs clarification.
5. In the same context, the paper cites Wikipedia with " *We must all* *accept that science is data and that data are science". *I have two problems with this: nobody must accept anything in science, all is matter of discussion; and this sentence is either trivial or it makes no sense. My advice would be to avoid these kind of sentences.
6. Later on, the paper presents "analogies" between RS and RD. Analogy, even if it exists, does not mean "similarity", and I think that this comparison is somehow misleading because the underlying assumption is not entirely correct ("a definition for RD can be proposed following the main features of the RS definition"). Software and data are different objects, with different issues (IP protection, communities etc.); the analysis of RS may be helpful for a better understanding of RD but this does not mean that both are more or less similar or even "fungible".
7. In section 3, I would suggest that the paper tries to describe the relationship between RS and RD, perhaps with "use cases". 
8. I admit that the authors are not legal experts but section 3 should be more explicit (and perhaps shorter and more restrictive) about the different laws and legal frameworks. Are you speaking about French laws? Or about the EU regulation?
9. Another, related issue is the data typology. The paper is about research data but section 3 mentions (and apparently does not differentiate) environmental data, cultural data and public sector information. 
10. My suggestion would be to improve the structure of section 3 and to distinguish between concepts, typology, legal status and reuse/policy (subsections).
11. Section 4: I already mentioned it above - RD is not only output but also input, with different issues (third party rights etc). This requires clarification.
12. At the end of section 4, the paper states that "documentation, licenses, Data Management Plans and other documents can also be part of the set of files that constitutes the RD". The meaning of this statement requires attention, as well as its implications. Does this mean that "RDM and other documents" are data? Or that they may be part of a RD deposit? But again (see above, comment 5), a statement that "all is data" is not helpful; it may make sense as a political catchword but not in an academic paper.
13. Last comment: I like very much Borgman's assessment of RD and her "conundrum challenges" but I have a somewhat different understanding of the meaning of this - for me, these "challenges" are questions that require attention and evaluation in a given situation, not for all RD in a general way. For me, they provide a kind of "reading grid" to analyse a specific data community, or a specific instrument or infrastructure or workflow; but they don't require or demand a comprehensive response as such provided by the paper.
14. Anyway, the paper is an interesting contribution to the academic research on RD, and I am looking forward to read a revised and improved version. Thank you!

Is the work clearly and accurately presented and does it cite the current literature?

Partly

If applicable, is the statistical analysis and its interpretation appropriate?

Not applicable

Are all the source data underlying the results available to ensure full reproducibility?

No source data required

Is the study design appropriate and is the work technically sound?

Partly

Are the conclusions drawn adequately supported by the results?

Partly

Are sufficient details of methods and analysis provided to allow replication by others?

Partly

Reviewer Expertise:

Information science

I confirm that I have read this submission and believe that I have an appropriate level of expertise to confirm that it is of an acceptable scientific standard, however I have significant reservations, as outlined above.

# Response

**Published:** 2022-04-22

**Authors:** Teresa Gomez-Diaz

**Affiliations:** LIGM, Gustave Eiffel University & CNRS, France

Many thanks to you, Joachim Schopfel, for your interesting comments that give us the opportunity to improve this work. A new version is in preparation, but we provide here some answers to your comments.

 1. [translations into English]

 Translations are included as footnotes, they will be moved to the main text.

 2. [information science (eg ISKO).]

 Many thanks for this reference, we are looking into it.

 3. [Open science is a fuzzy concept…]

 As indicated in the introduction: *A more transversal and global vision can be found in the ongoing work for the UNESCO Recommendation on Open Science [Reference 6]. See also [Reference 7]*. We will explain better this point.

 4. [the paper is in some kind limited or reduced to the aspect of "research output". Generally, in the research process, research software and research data are not only output but also tools (software) and input (data). This needs clarification.]

 In our view, each "research output" is a potential input for new research work. For example a RS can be a tool to manipulate data or an input for a new RS, this can be in the form of a component, or in the form of a new version done by the initial research team or another one. A RD can be used by other teams (as a tool) to understand some problem, it can be modified to produce a new RD, or it can be included as part of a larger data set, that can be as well a new RD. To better understand the production context is not, in our view, a limitation. But you are right, this point needs clarification.

 5. [cites Wikipedia with " *We must all accept that science is data and that data are science*".]

 Please note that this cited phrase comes from [Reference 4], and as indicated to Referee T. Koltay, we have chosen to do this reference in a slightly different manner as done in the Borgman’s work, where we have found it.

 6.1. [similarity/analogy]

 When consulting Cambridge English Learner’s Dictionary dictionary we find:

*analogy: a comparison that shows how two things are similar*

 6.2. [Software and data are different objects, with different issues (IP protection, communities etc.); the analysis of RS may be helpful for a better understanding of RD but this does not mean that both are more or less similar or even "fungible".]

 It is one of the intentions of the present work to show the differences between data and software form the legal point of view. While software finds a somehow clear and simple presentation (Section 2.1), data is much more difficult to grasp, as studied in Section 3. But this is not an obstacle to present an unified vision of RS and RD as research outputs, as we can see in the RS and RD proposed definitions. The fact that we can propose a similar formulation for both definitions allows us to propose similar dissemination and evaluation protocols as you can find in the article that follows this work [Reference 13]. The fact that we can deal with RS and RD in a similar way does not mean that they are similar.

 7. [describe the relationship between RS and RD, perhaps with "use cases".]

 It seems to us that it is quite usual for the targeted research audience to use and/or produce RS and/or RD as part of their everyday research practices, and that this point does not require further explanation. Examples can be found easily in the literature, as for example in the bibliography included at the end of this work.

 8. [I admit that the authors are not legal experts but section 3 should be more explicit (and perhaps shorter and more restrictive) about the different laws and legal frameworks. Are you speaking about French laws? Or about the EU regulation?]

 As indicated in the introduction, we have consulted legal texts and legal experts’ work in order to understand and explain the legal context in which we place this work. We have consulted French, European and USA texts, and selected the parts that we have used to document the article. We consider that our role is restricted to this intention, due to the lack or further expertise in legal matters, which does not hide the efforts we have put in to understand and to explain some legal issues. But we are unable to give more information on the regulations that can be taken into consideration, as this is the role of legal experts in the light of a well defined setting.

 9. [Another, related issue is the data typology. The paper is about research data but section 3 mentions (and apparently does not differentiate) environmental data, cultural data and public sector information.]

 The goal of Section 3 is to show the difficulties existing to set a data definition from the legal point of view, which is a very different context as the one existing for software, as shown in Section 2.1. The case of cultural data is very interesting, as legally speaking [Reference 19] *the quality of the producer legal entity defines the cultural quality of the data*. Then we can establish the parallel with the quality of research for some data set, as the consequence of the research quality of the producer team. Data typology could be the object of future work.

 10. [My suggestion would be to improve the structure of section 3 and to distinguish between concepts, typology, legal status and reuse/policy (subsections).]

 We will consider this suggestion

 11. [Section 4: I already mentioned it above - RD is not only output but also input, with different issues (third party rights etc). This requires clarification.]

 As already explained, we study in here the production aspects, and other aspects are presented in [Reference 13]. But you are right, this needs better explanation.

 12. [At the end of section 4, the paper states that "documentation, licenses, Data Management Plans and other documents can also be part of the set of files that constitutes the RD". ]

 Section 2.1 shows that the preparatory design work and documentation are part of the software, and these are documents that can be included in the released version of a RS, following the choice of the RS producer team. There can be other elements as for example tests, input and output files to illustrate how to use the RS, licenses, etc. To include these elements in the released RS correspond to best practices that facilitate RS reuse. In our view, to release a RD can follow similar practices, that is, to include a documentation, some use examples, a license, a data management plan…this is to be decided by the producer team.

 13. [Last comment: I like very much Borgman's assessment of RD and her "conundrum challenges" but I have a somewhat different understanding of the meaning of this - for me, these "challenges" are questions that require attention and evaluation in a given situation, not for all RD in a general way. For me, they provide a kind of "reading grid" to analyse a specific data community, or a specific instrument or infrastructure or workflow; but they don't require or demand a comprehensive response as such provided by the paper.]

 In our experience, Borgman's conundrum challenges correspond to questions that appear regularly at different stages of the RD production. We think that to provide such vision as the one exposed in Section 4 could be of help to deal with these questions, and, as you said, as a first step to tackle some problems in a well determined situation. Moreover, this view proposed in Section 4 is extended and completed with the dissemination and evaluation protocols proposed in [Reference 13]. Our experience of many years confirms the need of these protocols for RS, and we think that they will be appropriated, useful and relevant for RD as well.

 Teresa Gomez-Diaz and Tomas Recio

# Peer review: Reviewer response for version 1

**DOI:** 10.5256/f1000research.82187.r121511

**Published:** 2022-02-23

**Authors:** Remedios Melero

**Affiliations:** 1 Instituto de Agroquímica y Tecnología de Alimentos, CSIC, Valencia, Spain

The authors proposed a Research Data (RD) definition "based in three characteristics: the data should be produced (collected, processed, analyzed, shared & disseminated) to answer a scientific question, by a scientific team, and has yield a result published or disseminated in some article or scientific contribution of any kind." From my point of view this definition restricts RD to those that are published by a scientific team, however what about the citizen science, or data produced by non-scientist staff? What about any other data that do not deserve be published but help to further research?

 Authors say: "the RS is involved in the obtention of the results presented in scientific articles" - This is not necessarily true. RS is not always involved in the obtention of results because it can be developed for any other purpose, again the authors make a very strict definition.

 Authors say: "As a matter of fact, a research team can use RS produced by other teams for their scientific work, as well as FLOSS or other software developed outside the scientific community, but the present work is centered in the making-of aspects which are pertinent for the proposed definition." - This restricts the definition of Research Software (RS) a lot by excluding all FLOSS produced by non-academic members.

 The authors have missed any mention to the *Directive* (EU) 2019/1024 of the European Parliament and of the Council of 20 June 2019 on open data and the re-use of public sector information, in which RD are defined and included as part of the public sector. In fact, the authors have cited it but they have not commented/mentioned the fact that RD has a wider meaning and that according to this Directive are considered public sector information, and they need not necessarily be published in a scientific journal but shared.

 Definitions given by dictionaries are not particularly relevant to the scientific context/environment. I think this part should be omitted, it only adds some definitions in the authors' own languages.

 "For example, to the need for complementary, technical information associated to a given dataset in order to facilitate its reuse." - This is part of the FAIR principles which are not mentioned/linked to this comment. Obviously, a dataset without any information about how data have been produced/obtained, etc. are not valuable.

 Authors write: "In here, the research outputs have reach a status in which the research team is happy enough for its dissemination." - This seems a very naïve assertion. Because the authors "do not consider production issues like best software development practices or data curation", it seems they do not care about these important issues.

 Conclusions again repeat the proposal of a RD definition. Concepts like linked data, FAR data, and open data have not been treated in the article. Their definition of RD is very strict and narrow, and they have not considered any semantic issues about data and the benefits and implications of being a 5star open data. Their definition is far from the 4th or 5th step of the stars.

 In general, from my point of view, the article does not add any new ideas about RD definition and restricts it to data produced by scientific teams.

Is the work clearly and accurately presented and does it cite the current literature?

Partly

If applicable, is the statistical analysis and its interpretation appropriate?

Not applicable

Are all the source data underlying the results available to ensure full reproducibility?

No source data required

Is the study design appropriate and is the work technically sound?

Partly

Are the conclusions drawn adequately supported by the results?

Partly

Are sufficient details of methods and analysis provided to allow replication by others?

Partly

Reviewer Expertise:

Open science, open research data, scholarly publications, open access policies

I confirm that I have read this submission and believe that I have an appropriate level of expertise to confirm that it is of an acceptable scientific standard, however I have significant reservations, as outlined above.

# Response

**Published:** 2022-02-28

**Authors:** Teresa Gomez-Diaz

**Affiliations:** LIGM, Gustave Eiffel University & CNRS, France

Many thanks to you, Remedios Melero, for these very interesting comments. We are preparing a new version of this article and we will include several of the proposed corrections. Meanwhile, we would like to provide in here some preliminary comments.

 1. [this definition restricts RD to those that are published by a scientific team, however what about the citizen science, or data produced by non-scientist staff?]

 [the article does not add any new ideas about RD definition and restricts it to data produced by scientific teams.]

 It would be strange to consider any article published in a newspaper as a scientific publication.

 On the other hand, scientists may read the newspapers and many other documents, including tweets, and may use these documents as input information for a research work. As already explained in our answer to Rob Hooft’s comment, yes, we have chosen a restricted definition for RD. It allows us to provide the answers to the *Borgman’s conundrum challenges* that are in the Conclusion section. As far as we know, we have not found in the consulted literature the proposition of such kind of answers in this complete view. Moreover, as the RD definition finds a similar formulation as the RS one, we can also translate RS dissemination and evaluation protocols to RD [Reference 13]. Once we understand well the restricted context, it can be studied its extension and then see which are the answers to *Borgman’s conundrum challenges *and the dissemination and evaluation protocols that can be proposed in the extended context.

 The fact that we do not include e.g. public sector data as RD is different from the claim that these data cannot be used as input for a research work. As explained in section 3.2 of [Reference 13], these external data components should be correctly presented and referenced, and some can also fall in the category of RD.

 2. [RS is not always involved in the obtention of results because it can be developed for any other purpose, again the authors make a very strict definition.]

 [This restricts the definition of Research Software (RS) a lot by excluding all FLOSS produced by non-academic members.]

 You are right, this point should be explained better. To obtain a research result may involve the use of software (FLOSS or not FLOSS), the development of software to support some work or service, and the development of RS by the research team as explained in [References 3, 14]. Note that RS can be also disseminated as FLOSS, which is the usual practice in the work of T. Recio and in the research lab of T. Gomez-Diaz. This is also similar for data and RD, that can be disseminated as open data, as well as for publications and research articles as seen in the previous point.

 3. [Research data defined in the *Directive* (EU) 2019/1024 ]

 This definition was included in the preparation versions of the present article, and it will be included again in the new version in preparation, following your advice.

 4. [Definitions given by dictionaries]

 In the difficulties to explain easily the concepts of data and information we have ended in the consultation of several dictionaries, including some in English. Some of the found definitions, mainly in Spanish and French have attracted our attention and we have decided to included them in Box 1. This box can be easily skipped by readers not interested in these definitions.

 We prefer to leave the reading of the content of this box at the choice of readers.

 5. [FAIR and "For example, to the need for complementary, technical information associated to a given dataset in order to facilitate its reuse."]

 Please note that FAIR principles appear in the [Reference 55] dated 2016, while [Reference 36] that we have chosen to illustrate the need for complementary, technical information is dated 2012. Moreover, this is also related to the importance of context, that is explained in the OECD Glossary of Statistical Terms, with PDF and WORD download versions dated 2007 [ https://stats.oecd.org/glossary/download.asp]. On the other hand, FAIR principles are considered in the second part of this work [Reference 13], as they are related to dissemination issues. We will also mention them in the second version of this first part.

 6. ["In here, the research outputs have reached a status in which the research team is happy enough for its dissemination."]

 [authors "do not consider production issues like best software development practices or data curation", it seems they do not care about these important issues.]

 You are right, this point should be better explained in the new version of the article. It is not that we do not care about these important issues, as they are part of the 3 ^rd^ step of the proposed CDUR evaluation protocol for RS and RD, see sections 2.3 and 3.3 of [Reference 13].

 7. [Concepts like linked data, FAIR data, and open data have not been treated in the article. Their definition of RD is very strict and narrow, and they have not considered any semantic issues about data and the benefits and implications of being a 5star open data. Their definition is far from the 4th or 5th step of the stars.]

 Please note that FAIR data and open data are treated in [Reference 13]. We will include in the second version the mention of the 5star open data, many thanks for this reference.

 Teresa Gomez-Diaz, Tomas Recio

# Peer review: Reviewer response for version 1

**DOI:** 10.5256/f1000research.82187.r121519

**Published:** 2022-02-08

**Authors:** Tibor Koltay

**Affiliations:** 1 Institute of Learning Technologies, Eszterházy Károly University, Eger, Hungary

The content of the first two paragraphs of the paper (especially the first one) seems to be less appropriate, compared to the purpose of your paper. I would thus advise you to consider rewriting these paragraphs.

 Your practice of providing the cited texts in the original language (French or Spanish) and providing the translations of these passages only in the footnotes is unusual and may be not appropriate for a readership that probably reads and writes only in English, or is not familiar with Spanish and/or French texts. As I see it, if you would want to make a favour to your readers, who prefer French or Spanish, the solution could be reverse this order, i.e. putting the original texts into the footnotes.

**Other remarks**

 I think that it would be better if the following sentence would be changed as follows:

- “Indeed, as remarked by Hanson *et al*., we must all accept that science is data and that data are science… ^4^”

This regards not only the form of citing, but content, because this remark comes from Borgman’s Conundrum, cited in your paper a couple of times.

 You describe three main characteristics of RS:

- “the goal of the RS development is to do research. As stated by D. Kelly: it is developed to answer a scientific question ^32^,
- it has been written by a research team,
- the RS is involved in the obtention of the results presented in scientific articles (as the most important means for scientific exchange are still articles published in scientific journals).”

In general, these three claims are correct. However, the first one of them is a little awkward. I would thus change it to anything like “the goal of the RS development is to support research. As stated by Kelly, it is developed to answer a general, or a specific scientific question. Writing the software requires close involvement of someone with deep domain knowledge in the application area related to the question. ^32^”. Theses sentences however may prove redundant, because you provide a more complete definition:

- “Research Software is a well identified set of code that has been written by a (again, well identified) research team…If take this, linger definition only, the expression “(again, well identified)” should be deleted.

You write that “Indeed, there is a difference between the concepts of algorithm and software from the legal point of view, as there is a difference between the mere idea for the plot of a novel and the final written work.” This is a brilliant idea, although I believe that it should not be restricted to the legal point of view.

 In my view, it seems to be dangerous to write about copyright issues without being legal experts. Personally, I have only basic knowledge of copyright laws, so I cannot judge the correctness of all your argument. Fortunately, what you describe is also related to different issues.

 I do not see any further problems. Therefore, I will not enumerate passages that are correct and rather straightforward. My suggestion is however, that you carefully review you text in order to reach clarity of your argument.

Is the work clearly and accurately presented and does it cite the current literature?

Partly

If applicable, is the statistical analysis and its interpretation appropriate?

Not applicable

Are all the source data underlying the results available to ensure full reproducibility?

No source data required

Is the study design appropriate and is the work technically sound?

Partly

Are the conclusions drawn adequately supported by the results?

Partly

Reviewer Expertise:

Information science

I confirm that I have read this submission and believe that I have an appropriate level of expertise to confirm that it is of an acceptable scientific standard, however I have significant reservations, as outlined above.

# Response

**Published:** 2022-02-28

**Authors:** Teresa Gomez-Diaz

**Affiliations:** LIGM, Gustave Eiffel University & CNRS, France

Many thanks to you, Tibor Koltay, for these very interesting comments. We are preparing a new version of this article and we will include several of the proposed corrections. Meanwhile, we would like to provide in here some preliminary comments.

 1. [first two paragraphs]

 We have chosen to start in a ''light'' manner an article that can ask for some effort to be understood, this is our author’s choice. It is the reader’s choice to skip these two first paragraphs or to enjoy them, as this does not have any consequence for the understanding of the content of the article.

 2. [translations to English]

 We agree with you, the translations to English in the footnotes may hinder the fluent reading of this work, we will modify the presentation.

 3. [Hanson et al. Reference]

 You are right, we have found this reference in Borgman’s work, but we have consulted the original article and we have chosen to do this reference in a slightly different manner.

 4. [RS definition characteristics]

 We will modify the phrase to include your proposition as follows: “the goal of the RS development is to do or to support research’’. Please note that the composition of a research team involved in the

 development of a RS has been thoroughly studied in section 2.2 of [Reference 3]. We will include this reference to clarify this point as you ask. Please, also note that long developments may involve many different contributions from developers with different status. As copyright issues enter into play, it is important that the RS developers and contributors are correctly listed.

 5. [Algorithms and software]

 Comparisons between algorithms and software can be done in several contexts, for example in mathematics, or in computer science, among others. We have highlighted the legal aspects as we detect regularly the confusion between these two concepts, and the [Reference 22] providers a pretty clear explanation.

 6. [Copyright issues]

 Please note that one of the authors has study copyright issues in order to write [Reference 2], work that has been validated by several experts, including legal experts. On the other hand, we are regularly in contact and follow the work of legal experts, in such a manner as to provide us with the necessary confidence to deal with copyright issues in the way we propose in this article. The remark included at the end of the Introduction gives the necessary warning to our readers on this point.

 Teresa Gomez-Diaz, Tomas Recio
